# CD271 is an imperfect marker for melanoma initiating cells

**DOI:** 10.18632/oncotarget.1967

**Published:** 2014-05-13

**Authors:** Yann Cheli, Vanessa F. Bonnazi, Arnaud Jacquel, Maryline Allegra, Gian Marco De Donatis, Philippe Bahadoran, Corine Bertolotto, Robert Ballotti

**Affiliations:** ^1^ INSERM U1065, Equipe 1, Biologie et pathologies des mélanocytes: de la pigmentation cutanée au mélanome, Equipe labellisée Ligue 2013, Centre Méditerranéen de Médecine Moléculaire, Nice, France; ^2^ Université de Nice Sophia-Antipolis, UFR Médecine, Nice, France; ^3^ CHU Nice, Service de Dermatologie, Nice, France; ^4^ INSERM U1065, Equipe 2, Cell death, differentiation and cancer, Centre Méditerranéen de Médecine Moléculaire, Nice, France; ^5^ CHU Nice, Clinical Research Center, Nice, France

**Keywords:** Melanoma initiating cells, CD271, slow growing, MITF

## Abstract

Understanding the molecular and cellular processes underlying melanoma plasticity and heterogeneity is of paramount importance to improve the efficiency of current treatment and to overcome resistance to chemotherapy drugs. The notion of plasticity and heterogeneity implies the existence of melanoma cell populations with different phenotypic and tumorigenic properties.

Using melanoma cell lines and melanoma cells freshly isolated from patient biopsies, we investigated the relationship between ABCB5+, CD271+ and low-MITF, expressing populations that were reported to display melanoma initiating cell properties. Here, we showed that ABCB5+ and CD271+ populations poorly overlap. However, we found that the CD271+ population is enriched in low-MITF cells and expresses a higher level of stemness genes, such as OCT4, NANOG and NES. These features could explain the increased tumorigenicity of the CD271+ cells. The rapid conversion of CD271+ to CD271− cells in vitro demonstrates the plasticity ability of melanoma cells. Finally, we observed that the transient slow-growing population contains only CD271+ cells that are highly tumorigenic. However, the fast growing/CD271+ population exhibits a poor tumorigenic ability. Taking together, our data show that CD271 is an imperfect marker for melanoma initiating cells, but may be useful to identify melanoma cells with an increased stemness and tumorigenic potential.

## INTRODUCTION

Melanomas are very aggressive neoplasms renowned for their resistance to existing therapeutics. The most recent treatments targeting the mutated BRAF (BRAF^V600E^) have shown a spectacular high level of response, but in most cases melanomas acquire secondary resistances causing dramatic relapses [1].

Both primary and secondary resistances, as well as the exacerbated invasive properties of melanomas might be due to their remarkable phenotypic plasticity, allowing melanoma cells to adapt to different microenvironments and to chemotherapeutic drugs.

Cellular processes that underlie phenotypic plasticity are still a matter of debate between the proponents of the models of melanoma initiating cells and those of the phenotypic switch [5], even though, both models are not exclusive. The melanoma initiating cell model stems from the initial works of Lapidot et al [2] in leukemia, Singh et al [3] in brain cancer and Ricci-Vitani et al [4] in colon cancer. These pioneer works defined tumor initiating cells as cells responsible for tumor formation and maintenance, in vivo, in xenograft models.

Numerous reports showed that within a tumor or in cultured cells, all melanoma cells do not possess the same ability to form tumors when injected into nude or SCID mice. A small population is endowed with a high tumorigenic potential and has been designated as Melanoma Initiating Cells (MICs) [5, 6]. MICs showed an increased resistance to drugs and an exacerbated invasive and stemness phenotype [7, 8]. The existence of a reversible phenotypic switch has also been reported between these MICs and their less tumourigenic progeny [9].

However, no consensual marker characterizing the MICs population has been identified to date. High levels of CD24[10], ALDH[11], CD271[6], ABCB5[5] or JARID1B[12] have been reported in this population.

The MICs have also been characterized by a low MITF expression. Indeed, transient inhibition of MITF expression, which is considered as a melanoma oncogene [8, 13, 14], blocks melanoma cell growth in vitro, but counter-intuitively favors xenograft and metastasis development in nude mice. Further, depletion of the low-MITF population, which is a slow growing population, impairs severely the tumorigenicity of melanoma cells [9]. In agreement with these observations, two different groups, including ours, have shown that a transiently slow growing population has increased tumorigenic properties [9, 12].

CD271, also known as NGFR, is a receptor for the nerve growth factor (NGF), member of the neutrophin family proteins. NGFR has been shown to be specifically expressed on neural crest cells from which melanocytes are derived [15, 16]. Of note, several reports have shown that the CD271+ melanoma cells were endowed with a higher tumorigenic potential when injected into nude mice [6, 16]. Similarly, ABCB5, an ATP- dependant cassette transporter of various substrate such as small ions, sugars, peptides, and complex organic molecules [17], has been shown to confer doxorubicin resistance to melanoma cells [18]. Also, ABCB5 has been found at the surface of a subset of melanoma cells that exhibit enhanced tumor initiating cell phenotype [5]. However, no increased tumorigenicity has been observed when CD271+ or ABCB5+ melanoma cells were injected into NSG mice lacking NK Cells [16, 19].

To better characterize the population(s) of melanoma cells with a high tumorigenic capacity, we investigated the relationship between three of the markers mentioned above, i.e. CD271, ABCB5 and MITF.

Our data show that the CD271+ population is enriched in low-MITF and slow-growing cells that could account for the increased tumorigenicity of the CD271+ cells. Further, the CD271+ population has an increased level of stemness genes, such as OCT4, NANOG and Nestin. However, all the CD271+ cells have not the same tumorigenic potential. Indeed among the CD271+ cells, only the slow-growing population displays a high tumorigenic potential. Therefore, we conclude that CD271 is an imperfect melanoma initiating cell surface marker. However, in the absence of consensual surface marker for melanoma initiating cells, CD271 may be useful to identify melanoma cells with an increased stemness and tumorigenic potential.

## RESULTS

### Characterization of ABCB5 and CD271 populations in melanoma cell lines

**Figure 1:**
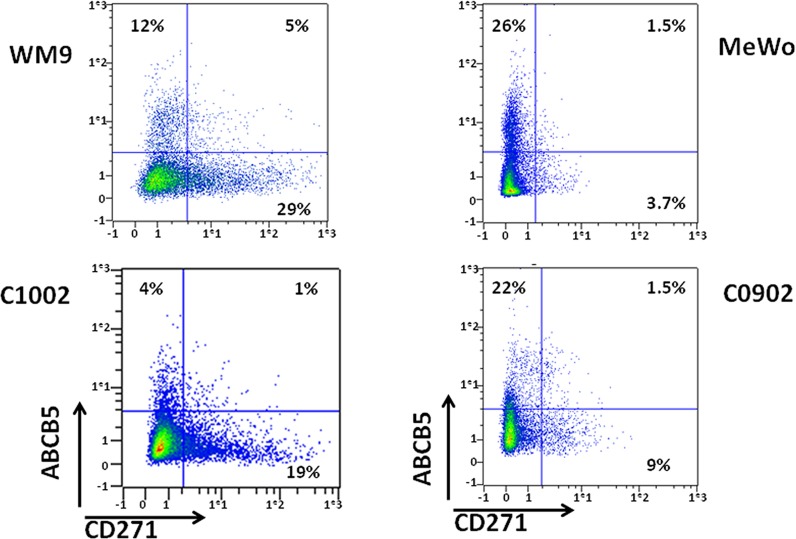
Analysis of ABCB5 and CD271 populations in melanomas WM9, MeWo, C1002 and C0902 were labeled for CD271 and ABCB5. Cells were analyzed by flow cytometry. CD271 intensity was plotted on abscissa and ABCB5 on ordinate.

First, we wanted to determine if two of the most studied Melanoma Initiating Cell (MIC) markers, ABCB5 and CD271 were shared by the same population or were expressed by different cell populations. To answer this question, 501-Mel, MeWo, WM9 and A375 cell lines, as well as three melanoma cell cultures established from patients (C0902, C1006, C1002), which are grossly representative of the mutational landscape in human melanomas, were stained with CD271 and ABCB5 antibodies. Flow cytometry analysis showed that the distribution of ABCB5+ and CD271+ populations varied in the different cell cultures, ranging from 5.2 to 75% for CD271+ and from 5 to 44% for ABCB5+ (figure 1, sup figure 1). The double positive population, ABCB5+/CD271+, differed also a lot, ranging from 1 to 29%. Interestingly, in 501-Mel cells, both populations overlapped clearly, as 90% of the CD271+ cells were also ABCB5 positive, while about 50% of the ABCB5+ population expressed CD271. In other cells a very poor overlap of the two populations has been observed, except in A375 cell line that does not express MITF. These results illustrate the phenotypic heterogeneity within a melanoma cell culture and between different melanoma cell cultures.

### CD271+ population is enriched in low-MITF cells

**Figure 2:**
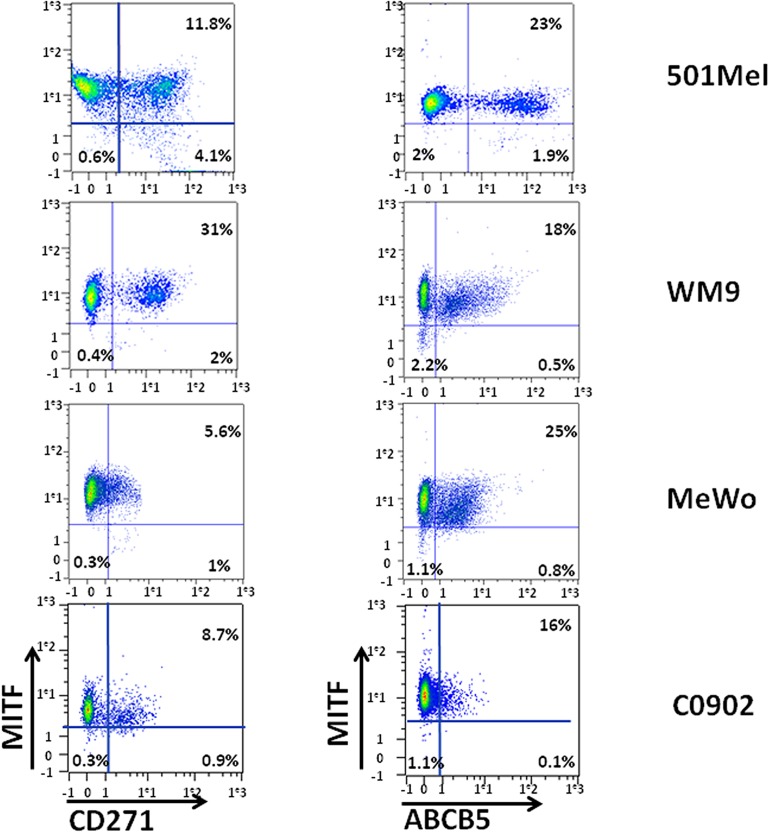
Analysis of MITF expression in CD271 and ABCB5 populations WM9, 501-Mel, MeWo and C0902 were labeled for CD271 and MITF (left panel) or ABCB5 and MITF (right panel). CD271 or ABCB5 intensity was plotted on abscissa, and MITF expression on ordinate.

We have previously shown that melanoma cell cultures contained a low-MITF population endowed with MIC properties [13]. We next wished to investigate whether the low-MITF population overlapped with the populations expressing CD271 or ABCB5 surface markers. To this aim, 501Mel, WM9, C0902, C1206 and MeWo cells were labeled with antibodies directed against CD271 and MITF or ABCB5 and MITF and analyzed by flow cytometry (figure 2, sup figure 2 A). In 501-Mel, the low-MITF population accounted for 3.9 to 4.7% of the cells and the vast majority of ABCB5+ and CD271+ cells had a high level of MITF expression. However, in this cell line, the percentage of low-MITF cells was markedly increased in the CD271+ population compared to the entire population (25.8% to 4.7%) (table 1). A slight increase in the low-MITF population has been observed in the ABCB5+ cells compared to the total 501-Mel cell population. In WM9, C0902, C1206 and Mewo cells, we also observed an increase in the low-MITF cells in the CD271+ population compared to the entire population (table 1).

**Table 1:** Percentage of low-MITF cells in the different melanoma cell populations

	total	ABCB5+	ABCB5−	CD271+	CD271−
501Mel	3.9-4.7	5.2	2.7	25.8	0.72
WM9	2.4-2.7	2.8	2.7	6	0.6
Mewo	1.3-1.9	3.2	1.3	15.2	0.3
C0902	1.2	0.7	1.3	9.4	0.33
C1206	2	5.1	1.87	22	1.24

**Figure 3:**
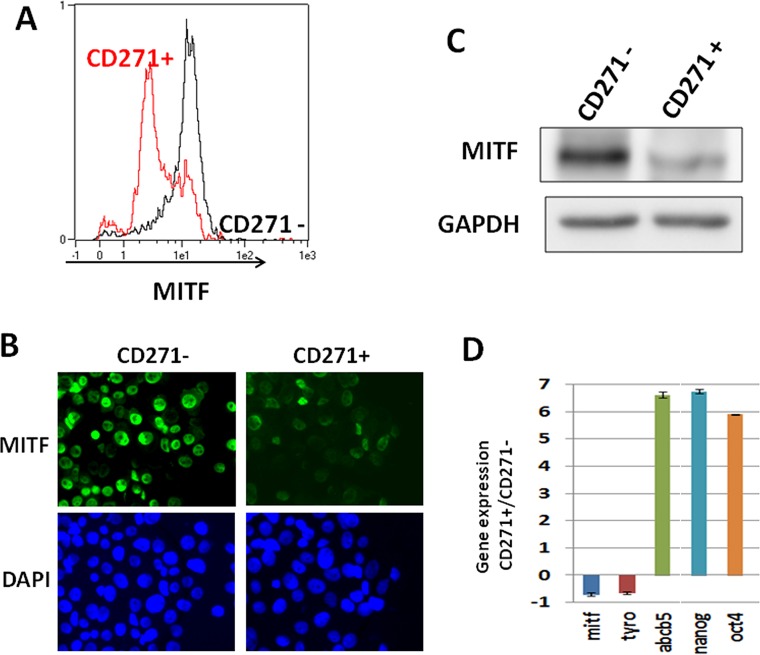
CD271 is enriched in the low-MITF population A) WM9 cells were sorted for CD271 expression (CD271− in black, CD271+ in red), then cells were fixed and stained for MITF expression. Intensity of staining was plotted in abscissa. B) WM9 cells sorted for CD271 expression were cytospined and then stained for MITF content. Cells were visualized by epifluorescence microscopy. Dapi was used to label nuclei. C) CD271+ and CD271− sorted cells were analyzed by western blot for MITF content. GAPDH was used as loading control. D) RNA from sorted cells for CD271 expression was analyzed by quantitative PCR. Stem cells (OCT-4, Nanog, ABCB5) and differentiation (MITF, TYRO) associated genes were quantified and normalized to SB34 as reference gene. Relative expression was determined by delta delta ct method. Values represent the mean+/− SD from three independent experiments

To confirm the enrichment in the low-MITF cells within the CD271+ population, we sorted the WM9 CD271+ population, and cells were stained for MITF and analyzed by flow cytometry (figure 3A). The CD271− population contained a vast majority of cells expressing a high level of MITF, while the CD271+ population was enriched in cells expressing low level of MITF. Immunofluorescence analysis of CD271+ and CD271- populations using MITF antibody confirmed that the CD271+ population was enriched in low-MITF cells (figure 3B).

Analysis of MITF protein expression by western blot, in both CD271+ and CD271− cells by western blot (figure 3C), demonstrated a decreased MITF expression in the CD271+ population. Finally, qPCR analysis of the mRNA content of CD271+ and CD271− populations showed a loss of differentiation markers such as MITF and tyrosinase, while the expression of MIC and stemness markers such as ABCB5, OCT4 and Nanog were markedly increased in the CD271+ population (figure 3D). The loss of differentiation markers such as MITF and tyrosinase in the CD271+ population has also been observed in other melanoma cells such as 501Mel and MeWo (Supp. figure 2B).

### Increased stemness gene expression in the CD271+ population

MICs have increased stemness features, evaluated by a higher level of stem cell genes. To evaluate the stemness status of the CD271+ cells, we analyzed the gene expression profile of CD271+ and CD271− populations, in both 501Mel and WM9 cells, using Stem Cell Pluripotency TaqMan® Low Density Array. Among the 96 genes evaluated (90 candidates and 6 controls), 14 were up-regulated in the CD271+ population in both 501-Mel and WM9 cells. Notably, the pluripotency genes *Lin28, POU5F1 (OCT4)* and *SOX2* were up-regulated, as well as *NES* a well known stem cell marker (table 2).

**Table 2:** Liste of genes regulated in the Stem Cell Pluripotency TaqMan® Low Density Array.

		Fold increase in CD271+ population, Log2						
		WM9		501Mel				
Gene							mean	p
EBAF		3,13	1,42	7,68	6,82		4,76	0,007
EEF1A1		0,84	0,55	2,86	3,56		1,95	0,035
FOXD3		3,00	−0,14	5,31	5,88		3,51	0,030
HBZ		2,37	−0,23	4,15	6,55		3,21	0,047
IFITM1		2,82	1,55	7,55	6,83		4,69	0,006
IFITM2		1,75	0,91	6,88	7,69		4,31	0,017
LEFTB		4,28	1,23	9,22	7,88		5,65	0,012
LIN28		4,00	1,23	8,81	7,76		5,45	0,011
NES		2,42	0,84	6,88	6,88		4,25	0,012
POU5F1		2,89	1,30	6,95	6,98		4,53	0,007
PTEN		2,70	1,29	5,86	5,60		3,86	0,004
SOX17		4,07	1,56	7,82	8,54		5,50	0,008
SOX2		2,32	0,99	7,37	8,01		4,67	0,015
TDGF1		3,61	1,05	8,54	7,86		5,27	0,013

Another characteristic of the cancer initiating cells is the increase in mesenchymal markers [21]. The expression of the fibronectin (FN1) transcript, a well known mesenchymal marker, was increased in the CD271+ population from WM9, but not from 501-Mel cells (not shown). The increased expression of FN1, and several stemness genes, such as SOX17 and LIN28a was confirmed at the protein level in the WM9, CD271+ population (supp figure 3). Therefore the increased expression of pluripotency, stemness and mesenchymal genes, in the CD271+ population, fitted well with an exacerbated tumorigenicity and the expected features of MIC.

### Biological properties and plasticity of the CD271+ and CD271− populations

**Figure 4:**
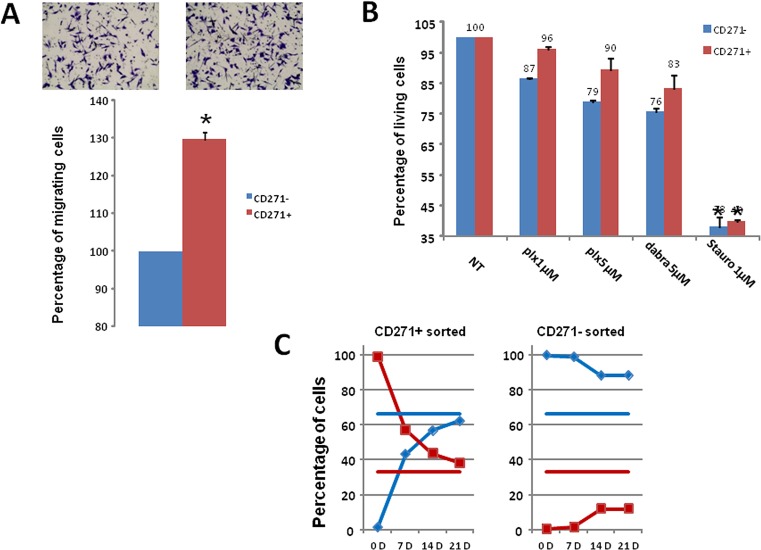
Analysis of CD271+ cells properties and plasticity A) CD271+/− cells were sorted then seeded in Boyden chamber for migration assay. Representative images and quantification of three independent experiments (mean+/−sd) are shown. B) Cell viability upon treatment with PLX4032, Dabrafenib, or staurosporine for 24 hours was assessed for CD271+ (red) or CD271− (blue) sorted cells. Results show the mean+/−SD from 3 independent experiments. C) CD271 Positive (left) and negative (right) WM9 cells were cultured for 21 days, and analyzed for CD271+ (red) or CD271−(blue) expression. Horizontal bars represent the percentage of CD271+ (red) or CD271− (blue) in the initial culture.

To evaluate the migration ability of the CD271+ and CD271− populations, WM9 cells were sorted according to CD271 expression. The same number of CD271+ and CD271- cells was then seeded in Boyden chambers for migration assay. CD271+ cells migrated slightly (30%), but significantly more rapidly than CD271− cells, showing a rather weak increase in this property (figure 4A). When evaluating the effects of BRAF inhibitors, CD271+ cells exhibited no significant increased viability compared to CD271- cells, after treatment with PLX04037 or Dabrafenib (figure 4B). Similar results were obtained in one patient cell culture, C1002 (sup figure 4). Therefore the CD271+ cells did not display a clear increase in the biological properties characterizing the MIC.

Then, we evaluated if subpopulation of a given phenotypic state (CD271+ or CD271) was able to return to the initial equilibrium proportion and reconstitute a mixed population identical to the original pool of cells from which they were extracted. When sorted CD271+ cells (98% pure, evaluated by analysis immediately after sorting) were cultured for 3 weeks, we observed progressive reemergence of the CD271− population until reaching a proportion of CD271+/CD271− cells (30%/70% respectively) comparable to that observed in the initial culture. Conversely, when purified CD271− cells (99% pure) were cultured, they failed to reconstitute the initial equilibrium proportion. Indeed, after 3 weeks, the culture contained only 15% of CD271+ (figure 4C), indicating that the transition from CD271− to CD271+ cells is a much slower process than the CD271+ to CD271− inter-conversion.

### Re-assessment of the tumorigenic potential of the CD271+ population

**Figure 5:**
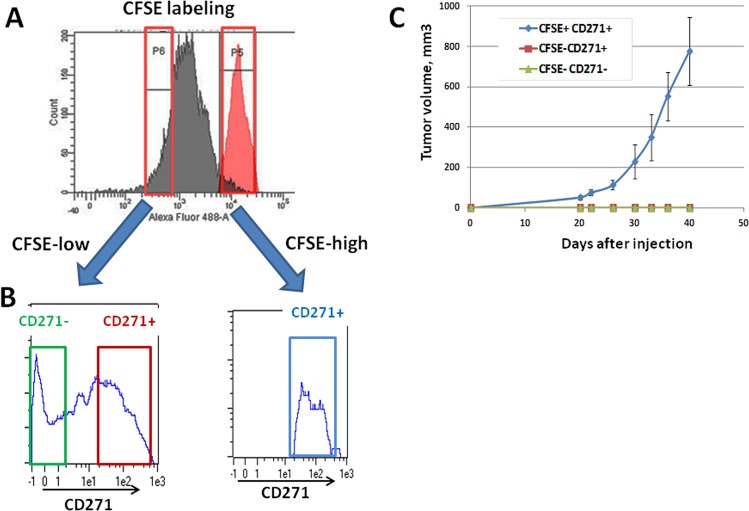
Analysis of the tumorigenicity of the different CD271 populations A) CFSE-labeled WM9 cells were grown for 72 h and sorted on a FACSAria for high-CFSE content (right part) and low-CFSE content (left part). In red is represented the charge of CFSE at day 0. B) CFSE-labeled cells were sorted, stained for CD271 and analyzed by flow cytometry on a MACSQuant cytometer. A representative histogram of CD271 content for each condition is displayed. C) CFSE-labeled WM9 cells were sorted and stained for CD271, then, CFSE-high/CD271+, CFSE-low/CD271+and CFSE-low/CD271−) were sorted and injected subcutaneously into nude mice. Mean+/−SEM of the tumor volumes is shown.

We have previously shown that a slow growing population, displayed a high tumorigenic potential and was enriched in low-MITF cells [9]. Therefore, we evaluated the expression level of CD271 in CFSE-low and CFSE-high populations, corresponding to fast and slow-growing cells respectively. Cells were labeled with CFSE and grown for 72 hours. After sorting of CFSE-high and CFSE-low populations (figure 5A), cells were labeled with CD271 antibody, and analyzed by flow cytometry. CD271 was expressed by both the slow (CFSE-high) and fast growing (CFSE-low) populations. However, the slow-growing population contained exclusively CD271+ cells (figure 5B). Since we have previously shown that the CFSE-high population was endowed with high tumorigenic abilities [9], it was expected that the CD271+/CFSE-low population was poorly tumorigenic.

To verify this hypothesis, the different populations CFSE-high/CD271+, CFSE-low/CD271+and CFSE-low/CD271−) were sorted as described above. 0.25×10^6^ cells of each population were injected subcutaneously into nude mice. Tumor formation was obtained with the CFSE-high/CD271+ population but not with CFSE-low/CD271+ or CFSE-low/CD271- cells (figure 5B). These observations indicate that all the CD271+ cells are not tumorigenic. Only the slow-growing sub-population, of the CD271+ cells, is endowed with high tumorigenic properties.

## DISCUSSION

In the recent years, resurgence of the “cancer stem cells” or “tumor initiating cells” theory has given rise to a plethora of studies that has fuelled controversy between the proponents and opponents to this hypothesis; especially in the field of melanoma. However, the controversy might be the consequence of a misleading interpretation of initial concepts introducing the notion of hierarchical organization in neoplasms [22]. Indeed, the cancer stem cell model is associated with hierarchical and uni-directional organization of the tumor, while the reversible organization is rather related to the phenotypic state transition [8, 23], both visions providing an explanation for the tumor heterogeneity. However, by no means, reversible and hierarchical organizations are antagonistic at both semantic and biological point of view. A hierarchy is not fixed and can be modified by external conditions. Therefore, both opinions can be easily reconciled.

The notion of tumor heterogeneity and the existence of cell subpopulations with specific tumorigenic properties are of paramount importance for the treatment of cancer. Indeed, it has been proposed that drugs target fast growing cells, sparing quiescent cell (cancer stem cells) leading to treatment failure and relapse. However, in most cases therapy fails not because it kills only proliferating tumor cells, but because it does not eliminate them [21]. This is the case for chemotherapeutic treatment of melanoma using alkylating agents such as dacarbazine. However, new therapies, targeting BRAF^V600E^ mutation, show a very high response rate, indicating that BRAF^V600E^ inhibitor eliminates proliferating melanoma cells. Unfortunately, very frequently patients rapidly relapse. This scenario can be explained by the pre-existence of specific clone bearing mutation conferring a resistance to the drug or to the existence of a melanoma cell population resisting to the treatment. The nature of this sub-population also needs also some clarification. The cells contained in this population have been qualified as tumor initiating cell based on their functional abilities to initiate and maintain tumor development. These cells that express some stemness markers, have self-renewal capacity and are quiescent can be considered as cancer stem cells. However, since these cells must become proliferative to allow tumor development, the notion of cancer stem-like cells has been introduced for defining cells combining both stemness (self-renewal) and tumorigenic (proliferation) properties [24, 25].

The surface markers identifying highly tumorigenic cells have been well characterized for hematological neoplasms [26], gliomas [27, 28] and breast cancer [27, 29]. In the case of melanoma, the lack of consensual surface markers has impeded greatly the biological and molecular characterization of melanoma initiating cells.

In the present study, we have analyzed the relationship between previously described melanoma initiating cells markers ABCB5, CD271 and MITF.

First, ABCB5 and CD271 appear to mark different melanoma cell populations, excepted in 501mel. Further, neither ABCB5 nor CD271 shows a correlation (or an anti-correlation) with MITF expression. This can be surprising since *ABCB5* has been described as a direct target of MITF. Indeed, over expression of MITF in human melanoma cells increase the expression of *ABCB5* and ChIP experiment showed that MITF binds to the *ABCB5* gene [30]. The low expression of ABCB5, in the vast majority of MITF-positive cells, indicates that ABCB5 is subjected to additional transcriptional or post-transcriptional regulations that remain to be identified.

Regarding CD271, it is worth remarking that flow cytometry, immunofluorescence and western blot analyses demonstrated that the CD271+ population is enriched in low-MITF cells. Furthermore, the CD271+ population expresses higher level of stemness markers, such as OCT4 and NANOG that are also upregulated in the low-MITF population [13]. Additionally, analysis of TaqMan Low Density Arrays confirmed the increased expression of genes associated with stemness and renewal such as *OCT4, NES*, *LIN28* and *SOX2*.

We also observed an increased expression of 3 genes involved in the NODAL pathway, *EBAF, LEFTB* and *TDGF1*. Several studies have documented a role of the NODAL pathway in melanoma progression and aggressiveness. Expression of TDGF1, also known as CRIPTO1, was previously described in melanoma [31-33]. Interestingly, the increased expression of EBAF and LEFTB, two NODAL inhibitors, concomitantly with a higher CRIPTO level, was described to sustain the undifferentiated stem cells phenotype [34]. For instance, CRIPTO activates the WNT pathway through LPR5/6 [35]. However, SOX17, which is also upregulated in the CD271+ cells, was reported to inhibit the WNT pathway [36-38]. Therefore, a fine tuning of the WNT and NODAL signaling pathway appears to be implemented in the melanoma initiating cells.

Besides, two receptors from the INF inducible transmembrane family (IFITM1 and IFITM2) are upregulated in CD271+ cells. This family of receptors was previously reported as potential markers in colon cancer [39] and is regulated by Wnt/beta catenin pathway, which is also deregulated in melanoma and involved in stem cells gene expression [40, 41]. Of note, IFITM2 expression is upregulated ensuing MITF silencing in melanoma cells (not shown) and deserves to be evaluated as a melanoma initiating cell surface marker.

Finally, attention should be paid to FOXD3, which plays a key role in neural crest cells fate determination. FOXD3 was reported to inhibit MITF expression [42], and therefore might participate to the maintenance of the melanoma initiating cell phenotype.

As a whole, these observations indicate that the CD271+ population is endowed with increased stem cell transcriptomic features. However, this population does not display the full spectrum of stem cell biological properties. Indeed, the CD271+ cells display only a slight increase in motility and no significant change in the response to BRAF inhibitors.

Another key characteristic of melanoma initiating cells is a transient slow growth rate [12, 13]. Interestingly, the slow growing melanoma cell population contains exclusively CD271+ cells, but approximately half of the CD271+ cells are fast-growing melanoma cells. The CD271+/slow-growing population has a high tumorigenic potential compared to the CD271+/fast-growing population. Therefore, all the CD271+ cells have not exacerbated tumorigenic properties, but the CD271+ population contains high level of low-MITF and slow-growing cells. This enrichment can provide an explanation for the increased tumorigenicity of the CD271+ cells previously reported [6, 16]. CD271 can be considered, *per se*, as an imperfect melanoma initiating cell marker. However, cancer initiating cells in other neoplasms have been characterized by at least 2 different surface markers; CD44+/CD24-/lin- in breast cancer, a2b5+/CD133- in glioblastoma and CD34+/CD38− in leukemia: [43]. Therefore the combination of multiple surface markers that will identify precisely the melanoma initiating cells still needs for further characterization.

Nevertheless, the original theory suggested that tumor development might be a caricature of normal tissues development, in which stem cells played a central role [44]. It was proposed that processes involved in normal tissues development would be targeted by oncogenic events and consequently would allow tumor development.

MITF plays a crucial role in melanocyte development by controlling growth and differentiation in this lineage. MITF itself is the target of a mutation that favors melanoma development [30, 45]. Furthermore, MITF is downstream the MAP Kinase pathway [46, 47] that is frequently deregulated in melanoma, due to BRAF^V600E^ mutation [48, 49]. MITF also functions downstream the PI3 kinase pathway [50] that is also deregulated in melanoma [51]. Therefore MITF, which is recurrently targeted by oncogenic events in melanomas, is the perfect candidate to regulate the transition between melanoma initiating cells and their more differentiated progeny.

The cells expressing low levels of MITF have a more stem cell-like phenotype, with an increased plasticity favoring thereby tumorigenicity. These cells are expected to express specific surface markers which expression should be inversely correlated to MITF. However, until now, such surface markers could not be found, maybe because random noise in gene expression can stochastically influence cell fate and regulation of the phenotypic equilibrium within populations of cells [7, 52].

Even though, CD271 is not a perfect marker for melanoma initiating cells, the CD271+ population is endowed with increased tumorigenic properties in vitro and in xenograft. Whether the CD271 population plays a role in melanoma tumor development, metastasis dissemination or in drug resistance will require a careful analysis of pathologic samples, up to single cell level. As we now have the technical ability to perform such approach, we may optimistically consider characterizing the melanoma initiating cells and developing an efficient anti-melanoma therapy targeting this population.

## MATERIAL AND METHODS

### Cell culture

Cells (A375, 501Mel, WM9, MeWo) were grown in Dulbecco's modified Eagle's (DMEM) medium (Invitrogen, Carlsbad, CA, USA) supplemented with 7% FCS and penicillin/streptomycin (100 U/ml/50 *μ*g/ml) at 37°C and 5% CO_2_. Patient melanoma cells (C0902, C1006, C1206, C1002) were prepared as described. Briefly, biopsy was dissected and digested for 1–2 h with collagenase A (0.33 U/ml), dispase (0.85 U/ml) and Dnase I (144 U/ml) with rapid shaking at 37°C. Large debris were removed by filtration through a 70-*μ*m cell strainer. Viable cells were obtained by Ficoll gradient centrifugation and cultured in RPMI medium (Invitrogen) complemented with 7% FCS and penicillin/streptomycin [20]. The mutational status of the cells is summarized in supplemental table 1.

### Antibodies and reagents

Antibody against GAPDH was purchased from Santa Cruz Biotechnology (Santa Cruz, CA, USA), anti-CD271 from BD Biosciences (Franklin Lakes, NJ, USA), anti-MITF (C5) from Abcam (Pleasanton, CA, USA), ABCB5 antibody from Rockland (Gilbertsville, PA). Secondary antibody (alexa 488 and alexa 647) and CellTrace CFSE cell proliferation kit were purchased from Invitrogen (Grand Island, NY, USA).

### Flow cytometry and fluorescence-activated cell sorting

For surface receptor staining, cells were detached with 2mM EDTA in phosphate-buffered saline (PBS), centrifuged, incubated for 30 min with primary antibodies in PBS 1% (W/V) bovine serum albumin (BSA), and then washed with PBS/EDTA. When necessary, cells were subsequently incubated with secondary antibodies (1/500 dilution) in PBS/BSA for 30min, washed and analyzed with MACSQuant analyzer (Miltenyi biotech, Bergisch Gladbach, Germany). For double staining, labeling with CD271 or ABCB5 antibody was performed as described above, and then cells were subsequently fixed in 3% paraformaldehyde for 10 min, permeabilized with 0.5% Triton-X-100 and stained with MITF specific antibody. Isotype-matched mouse antibodies were used as control. MITF was detected using anti-mouse Alexa Fluor 488-conjugated antibodies.

For *in vitro* CFSE assay, cells were labeled with 2 mmol/l of CFSE according to the manufacturer's protocol (Invitrogen), and then plated for 72 hours. Cells were then detached and stained with CD271 or ABCB5 antibodies as described above.

Cell sorting was performed using a FACSAria flow cytometer (BD biosciences, San Jose, CA, USA).

### Immunofluorescence labeling

After sorting with CD271 antibodies, cells were cytospined on a slide. MITF staining was performed as previously published [13]. Slides were analyzed by microscopy (Leica DM 5500B).

### Cell viability test

After sorting, cells were cultured for 6 hours before drugs were added for 24h at different concentrations. Viability was assessed using the Cell Proliferation Kit II (XTT; Roche Diagnostics, Meylan, France) according to the manufacturer's recommendations, and results were expressed as percentage of the value of DMSO-treated cells.

### Cell migration assay

The assay was carried out using the Cell Migration Assay kit (Chemicon International, Temecula, CA, USA). In brief, sorted cells were counted and allowed to migrate for 24–48h at 37ΰC in 5% CO_2_. The lower compartment of the chamber was filled with culture medium containing 7% fetal bovine serum. Cells at the lower membrane surface were fixed inPBS, 1% paraformaldehyde, stained with 0.1% crystal violet and counted (five random fields/well).

### Reverse transcription and Quantitative Polymerase Chain Reaction

Total cell RNA was extracted using the RNeasy miniprep kit (Qiagen), and 1μg of RNA was reverse amplified with oligo dT using reverse transcription system (Promega), according to manufacturer's instructions. PCR was performed using StepOnePlus real time PCR system, and the power SYBR green PCR master mix reagent (Applied biosystems, Foster city, CA). Relative quantification of the amplicons was performed by 2(-Delta Delta CT) method. Primer list details are available on request.

RNAs from CD271+ sorted-cells were also analyzed with the Stem Cell Pluripotency TaqMan® Low Density Array (TLDA) from Life Technologies according to the manufacturer's recommendations. Relative quantification of the amplicons was performed by 2(-Delta Delta CT) method.

### Statistics

Statistical analysis was performed using the Student's *t*-test. *p*<0.05 was accepted as statistically significant.

## SUPPLEMENTARY MATERIAL AND FIGURES


