# Supplementary material for: CD271 is an imperfect marker for melanoma initiating cells
Source: Oncotarget. 2014 May 13;5(14):5272–83. doi: 10.18632/oncotarget.1967 (PMC4170612; doi:10.18632/oncotarget.1967)
Supplement: Supplementary file 1 [file oncotarget-05-5272-s001.pdf]

CD271 is an imperfect marker for melanoma initiating cells

Supplementary Material

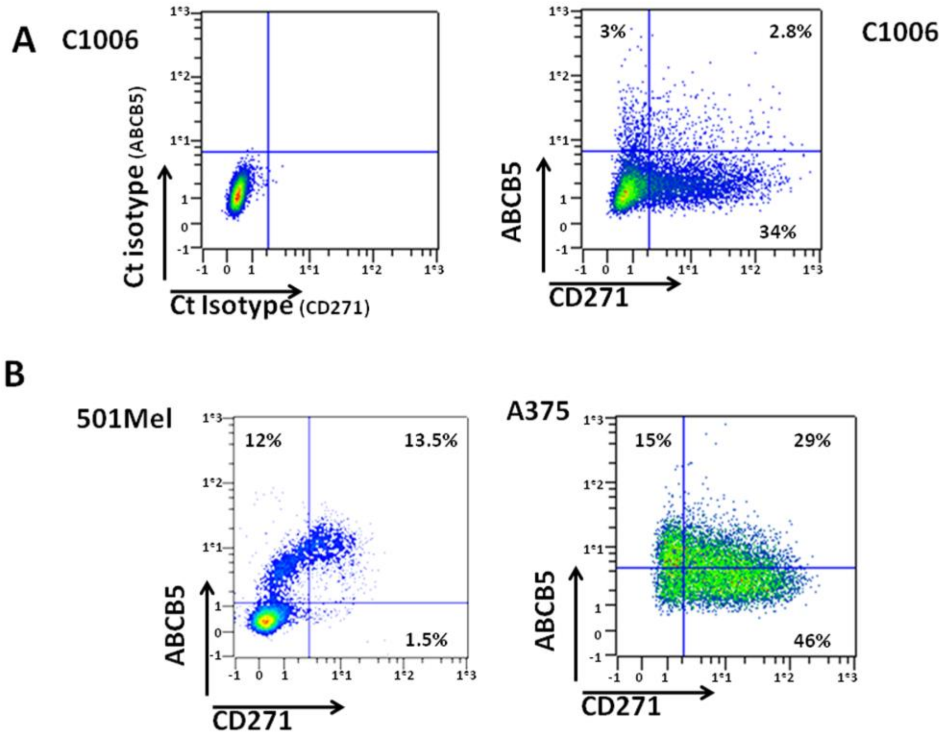

supp. figure1: Analysis of ABCB5 and CD271 populations in melanomas. C1006, 501Mel, and A375 cells were labeled for CD271 and ABCB5. Cells were analyzed by flow cytometry. CD271 intensity was plotted on abscissa and ABCB5 on ordinate. The upper left panel represents the labeling with matched control isotype antibodies.

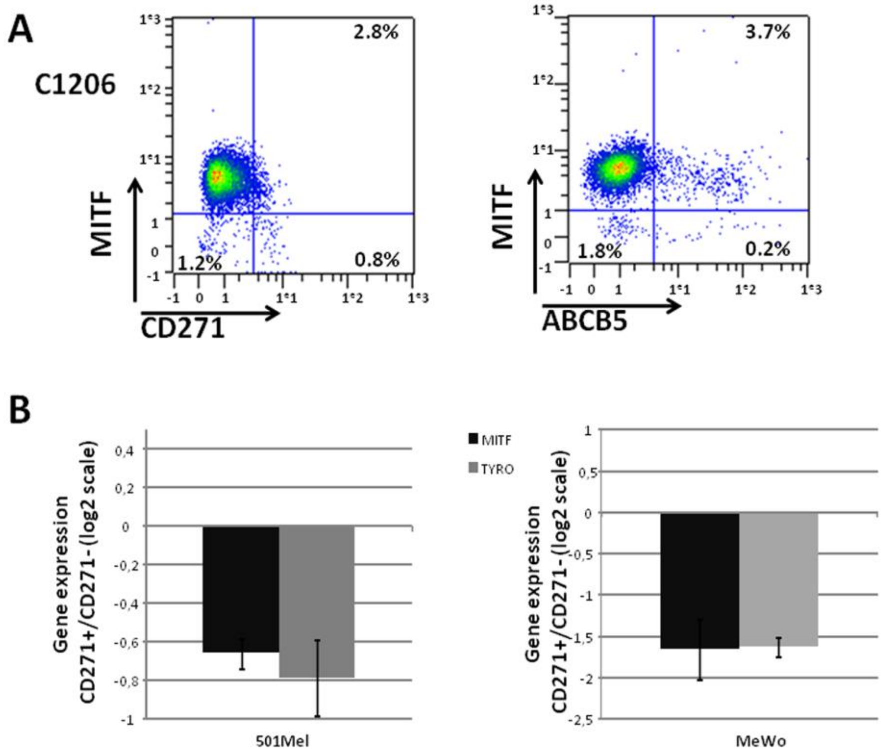

supp. figure2: Analysis of MITF expression in CD271 and ABCB5 populations. A) C1206 cells were labeled for CD271 and MITF (left panel) or ABCB5 and MITF (right panel). CD271 or ABCB5 intensity was plotted on abscissa, and MITF expression on ordinate. B) RNAs from CD271-sorted 501Mel and MeWo cells were analyzed by quantitative-PCR. Differentiation genes (MITF, TYRO) were quantified normalized to SB34 as reference gene. Relative expression to CD271- cells was determined by delta delta ct method. Values represent the mean+/- SD from three independent experiments

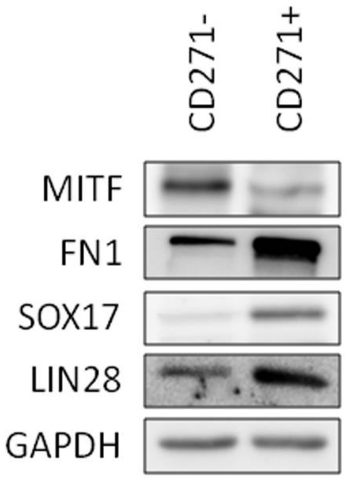

supp. figure3: Analysis of protein expression in CD271 populations. CD271 sorted 501-Mel cells were analyzed by western blot for MITF, FN1, SOX17, LIN28 content. GAPDH was used as loading control.

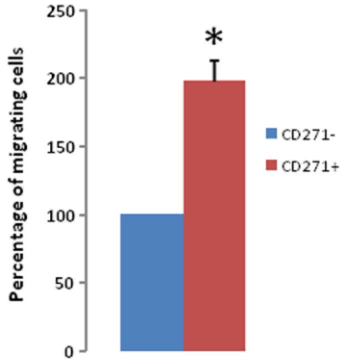

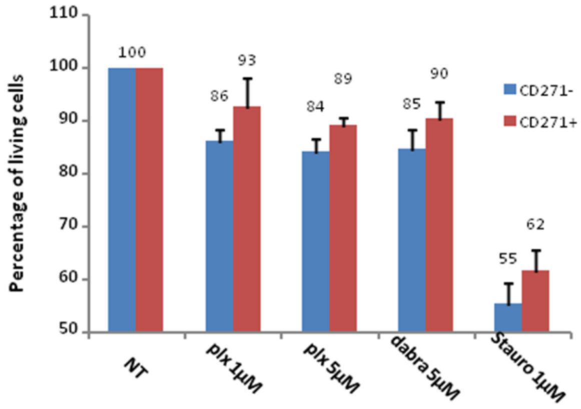

supp. figure4: Analysis of properties of CD271+ cells from patient. C1002 cells were sorted for CD271 and then seeded in Boyden chamber for migration assay. Representative images and quantification of three independent experiments (mean+/-sd) are shown. B) Cell viability upon treatment with PLX4032, Dabrafenib, or staurosporine for 24 hours was assessed for CD271+ (red) or CD271- (blue) sorted cells. Results show the mean+/-sd from 3 independent experiments.
